# Genome-Wide Analysis of DNA Methylation in Buccal Cells of Children Conceived through IVF and ICSI

**DOI:** 10.3390/genes12121912

**Published:** 2021-11-28

**Authors:** Bastien Ducreux, Jean Frappier, Céline Bruno, Abiba Doukani, Magali Guilleman, Emmanuel Simon, Aurélie Martinaud, Déborah Bourc’his, Julie Barberet, Patricia Fauque

**Affiliations:** 1 Equipe Génétique des Anomalies du Développement (GAD), Université Bourgogne Franche-Comté, INSERM UMR1231, 2 Rue Angélique Ducoudray, F-21000 Dijon, France; bastien.ducreux@outlook.fr (B.D.); jean.frappier@chu-dijon.fr (J.F.); celine.bruno@chu-dijon.fr (C.B.); magali.guilleman@chu-dijon.fr (M.G.); emmanuel.simon@chu-dijon.fr (E.S.); aurelie.martinaud@chu-dijon.fr (A.M.); julie.barberet@chu-dijon.fr (J.B.); 2 Laboratoire de Biologie de la Reproduction—CECOS, CHU Dijon Bourgogne, 14 Rue Gaffarel, F-21000 Dijon, France; 3 Faculté de Médecine, Sorbonne Université, UMS 37 PASS Plateforme P3S, 91, Bd de l’hôpital, F-75634 Paris, France; habiba.doukani@sorbonne-universite.fr; 4 Service de Gynécologie-Obstétrique, CHU Dijon Bourgogne, 14 Rue Gaffarel, F-21000 Dijon, France; 5 Institut Curie, PSL University, CNRS, INSERM, 26 Rue d’Ulm, F-75248 Paris, France; deborah.bourchis@inserm.fr

**Keywords:** assisted reproduction, children, DNA methylation, methylation array, culture medium

## Abstract

Early life periconceptional exposures during assisted reproductive technology (ART) procedures could alter the DNA methylation profiles of ART children, notably in imprinted genes and repetitive elements. At the genome scale, DNA methylation differences have been reported in ART conceptions at birth, but it is still unclear if those differences remain at childhood. Here, we performed an epigenome-wide DNA methylation association study using Illumina InfiniumEPIC BeadChip to assess the effects of the mode of conception on the methylome of buccal cells from 7- to 8-year-old children (48 children conceived after ART or naturally (control, CTL)) and according to the embryo culture medium in which they were conceived. We identified 127 differentially methylated positions (DMPs) and 16 differentially methylated regions (DMRs) (FDR < 0.05) with low delta beta differences between the two groups (ART vs. CTL). DMPs were preferentially located inside promoter proximal regions and CpG islands and were mostly hypermethylated with ART. We highlighted that the use of distinct embryo culture medium was not associated with DNA methylation differences in childhood. Overall, we bring additional evidence that children conceived via ART display limited genome-wide DNA methylation variation compared with those conceived naturally.

## 1. Introduction

Assisted reproductive technologies (ARTs) have been in use for more than 40 years. They are clinically effective thanks to the improvement of diverse procedures, such as conventional in vitro fertilization (IVF) or intracytoplasmic sperm injection (ICSI). Now that ARTs are commonly used, it is estimated that children born by ART worldwide represent approximately 4% of all births [1].

However, rising concerns about the absolute safety of these techniques appeared in the early 2000s. Major adverse perinatal outcomes were first reported following ART including increased risk of preterm birth, and low and very low birthweight [2,3]. Epidemiological studies with more extensive follow-up have now been performed, and they support that the majority of ART-conceived children are healthy, even though higher cardiometabolic risk profiles exist in ART offspring [4,5,6]. Potential long-term health risks, including malignances, associated with ART are still unknown in humans and might not be negligible [6,7].

Furthermore, there is an increasing awareness about the potential consequences of IVF/ICSI on a number of complications potentially linked to epigenetic deregulation, such as Beckwith–Wiedemann and Silver–Russell syndromes [8,9]. It has been suggested that prenatal and early life exposure to a stressful environment could affect developmental trajectories via epigenetic mechanisms [10,11,12]. Indeed, epigenetic processes control numerous major cellular functions that occur during development, including changes in gene expression directed by epigenetic marks (notably DNA methylation). Reprogramming of the epigenome is also essential for genomic imprinting and for the control of repeated sequences, which are major factors regulating development and growth of the conceptus. Specifically, a partial erasure of parental DNA methylation occurs during early embryogenesis, sparing imprinted controlled regions, followed by a remodeling of the methylome landscape [13] that persists into adulthood [14,15]. In ART, additional periconceptional exposure occurs during epigenetic reprogramming (controlled ovarian hyperstimulation, embryo culture medium), with the potential to alter DNA methylation set up [16].

There have been continuous efforts to improve the culture media used for preimplantation embryos, with the primary goal of increasing live birth rates [17]. Despite these enhancements, numerous studies using mouse models, including ours, provide reliable and relevant findings concerning the impact of the culture medium composition on epigenetic regulation [18,19,20,21,22]. Furthermore, the type of culture medium used in human reproduction may influence the phenotypic characteristics (such as birthweight) of children born after IVF [23,24,25,26]. However, recent studies in placenta [27] and buccal cells [28] found no differences in the DNA methylation profiles of imprinted genes of ART-conceived children for whom different culture media were used. In spite of this, there is still an assumption that the culture media used has an overall influence on the human embryo epigenome [29].

It is clear that the resulting epigenetic profile arising from ART could affect development later in life and predispose to adult-onset diseases. Moreover, these epigenetic modifications may be transmitted to further generations [30], especially on sequences that do not undergo epigenetic reprogramming during gametogenesis [31]. As considerable evidence in animals indicates that the ART themselves can negatively affect epigenetics [7], the safety of ART at the epigenetic level is still not accepted [32].

In humans, DNA methylation at imprinted genes has been intensively studied, which has highlighted that ART are likely to increase imprinting methylation errors [33]. For instance, on several occasions, our team found DNA methylation changes associated with imprinted genes in ART children as compared with naturally conceived children [28,34]. We also investigated DNA methylation levels in repetitive elements in ART children in comparison with naturally conceived newborns [34] and found differences in LINE-1 elements. Nevertheless, all regions are susceptible to DNA methylation changes, and only a very small number of epigenome-wide association studies (EWASs) of ART offspring were performed to date in humans, resulting in contradictory findings. Some studies found large [35] or low differential methylation [36,37] between ART and naturally conceived neonates in cord blood. On the other hand, Choufani et al. [38] and Gentilini et al. [39] did not find significant DNA methylation differences between ART-conceived and naturally conceived children in placenta tissue and cord blood, respectively. Finally, the largest EWAS to date found that, in blood, the differential methylation observed in ART neonates was minimized in childhood [40] and adulthood [41]. Given the rising number of ART-born children, it is essential to assess the safety of such techniques on the whole epigenome and at different ages.

To address the gaps in knowledge, we performed an epigenome-wide association study assessing more than 740,000 CpGs and CpHs using EPIC BeadChip on ART and non-ART children (aged 7.7 ± 0.7 years old), and we evaluated the impact of the culture medium composition. ART children were conceived following the use of two distinct culture media, one of which turned out to be significantly underperforming in the matter of preimplantation embryo development [42] and is no longer used. This investigation offers new insights into how ART and the culture medium remodel the epigenome in childhood, and the functional consequences.

## 2. Materials and Methods

### 2.1. Study Population

This single-center cohort study was based on an earlier randomized study conducted in 2008 at the University Hospital of Dijon with the primary aim of comparing outcomes following the use of two media: Global medium (LifeGlobal, Guelph, ON, Canada) and Single Step Medium (SSM, Irvine Scientific, Santa Ana, CA, USA). Singletons conceived in the randomized study were included in a previous study in order to collect medical data for the gestational, neonatal, and childhood period [42]. This former study was prematurely stopped 6 months after the start because the SSM medium significantly underperformed in preimplantation embryo development and pregnancy rates. The parents of all singletons from the Bouillon population (*n* = 73) were approached after the seventh birthday of their child to participate in an additional epigenetic study. In total, after parental consent, buccal smears of 37 singleton births were obtained for epigenetic analyses. For controls, naturally conceived singleton children born in the same period (between September 2008 and September 2009) and geographic region (Burgundy) as the ART children were included. Children were excluded from the naturally conceived group if there was any parental history of infertility or fertility treatment. One sample from the ART group was finally excluded for technical reasons (insufficient quantity of DNA). Epigenome-wide methylation data was analyzed using the EPIC array for 36 children (23 and 13 in the Global and SSM groups, respectively). Among these, 15 children were conceived using fresh embryo transfers after standard IVF (10 and 5 in the Global and SSM groups, respectively) and 21 after ICSI (13 and 8 in the Global and SSM groups, respectively). DNA methylation status was generated by EPIC array for 12 CTL children matched for gender and age. Participating children’s characteristics can be found in Supplementary Materials File S1.

### 2.2. Sample Preparation and DNA Methylation Extraction

As previously described, buccal smear samples were collected with the Oragene DNA Collection kit (OG-250, Genotek, Ottawa, ON, Canada) [28]. We extracted DNA with the Gentra Puregene Blood Kit (Qiagen, Valencia, CA, USA) according to the manufacturer’s protocol. For each sample, DNA quantity and quality was evaluated on a Nanodrop Spectrophotometer (ThermoFisher Scientific, Illkirch, France). We then performed bisulfite conversion using the EZ DNA Methylation Kit (Zymo Research, Orange, CA, USA) following the manufacturer’s instructions and methylome was assessed with the state-of-the-art Infinium HumanMethylationEPIC BeadChip (Illumina, San Diego, CA, USA). Finally, chip processing was performed using an Illumina HiScan SQ fluorescent scanner at the UMS 37 PASS platform (Sorbonne University, Paris, France). Arrays were visualized and analyzed using GenomeStudio v.2011.1 (Illumina, San Diego, CA, USA).

### 2.3. Statistical Analyses

Raw IDAT files obtained from methylome array data were pre-processed using the *MissMethyl* [43] and *minfi* [44] R packages. Sample quality was first checked before overall data was normalized using the Subset-quantile Within Array Normalization method [45] to remove technical variance between probe designs. Probes with a poor detection p-value (*p* < 0.01) were removed from the analysis, as were those associated with SNPs and cross-reactive probes. Because cell-type composition is often a major confounder in EWAS, cell-type proportions were predicted at this step with the recent deconvolution algorithm *HEpiDISH* [46]. Briefly, *HepiDISH* uses robust partial correlation to estimate cellular proportions in epithelial tissues according to the methylation levels of highly specific CpGs of epithelial cells and seven leukocyte subtypes. As repetitive elements and imprinted genes were already analyzed by our group for the same samples in a previous study for [28], we decided to remove them for this analysis. As a result, we used *REMP* package [47] to identify probes located within repetitive elements and removed them. We also removed probes located within a list of 76 imprinted genes established by Pervjakova et al. [48]. This left a total of 740,869 probes for downstream analysis. For each probe, a ß-value was calculated applying the following formula: ß = intensity of the methylated allele/(intensity of the methylated allele+intensity of the unmethylated allele + 100). ß-values are an estimator of methylation proportion at a given loci, with 0 meaning completely unmethylated and 1 fully methylated.

Singular value decomposition was next used to identify the following biological and technical confounders: Gender, Array position, Cell fractions of epithelial cells/B-lymphocytes/monocytes/neutrophils/natural killers (*p*-value < 0.05, Supplementary Materials File S2). We performed a differential methylation analysis using linear regression with the *limma* package (Smith, 2005) after converting ß-values to M-values, which possess more valid statistical properties to carry out differential analysis [49]. We incorporated the previously identified covariates in the final model and tested it with and without cell-type proportions. Differentially methylated positions (DMPs) associated with variables of interest were probes showing an adjusted *p*-value < 0.05 (Benjamini–Hochberg). A Δβ was calculated for each probe as the β-value difference between the two groups compared. The *DMRcate* method [50] was then used with default parameters to identify differentially methylated regions (DMRs). DMRs were those that contained at least 2 DMPs (FDR < 0.05). Probe annotations were retrieved from *ChAMP* package EPIC array annotation file [51]. A gene ontology enrichment test of DMRs was performed using the GOregion function from *MissMethyl* package. Association between genes containing DMR and diseases were tested in the DisGeNET database [52], a useful platform that computes gene–disease association scores taking into account, inter alia, the number of studies that reported the association in the literature. In addition, for each DMP found, in order to determine their potential involvement in conditions, we checked for any overlaps in the EWAS catalog and the EWAS atlas [53,54].

### 2.4. Comparison with Previous Studies

The location of DMPs and DMRs was compared with the results of 15 other studies on genome-wide DNA methylation and ART [35,36,37,38,39,40,41,55,56,57,58,59,60,61,62]. Genomic coordinates of DMPs and DMRs found in each individual study were extracted and annotated to the nearest gene. Finally, we recorded genes that contained DMP or DMR in both our study and at least one of the 15 studies included in the comparison.

## 3. Results

We investigated whether ART procedures could affect the buccal cell methylome in non-imprinted and non-repetitive element regions, seeing as imprinted genes and repetitive elements were already explored in this dataset [28]. We performed differential methylation analysis at a single CpG resolution in order to make comparisons between 36 ART-conceived children and 12 naturally conceived children.

Before assessing the impact of mode of conception (ART vs. CTL), type of culture medium, and method of fertilization, we analyzed the role of the cell composition in our samples.

### 3.1. Raw Analysis of the Buccal Cell DNA Methylation Profile of ART Children Reveals Major Variations in Cell Type Proportions

**Figure 1:**
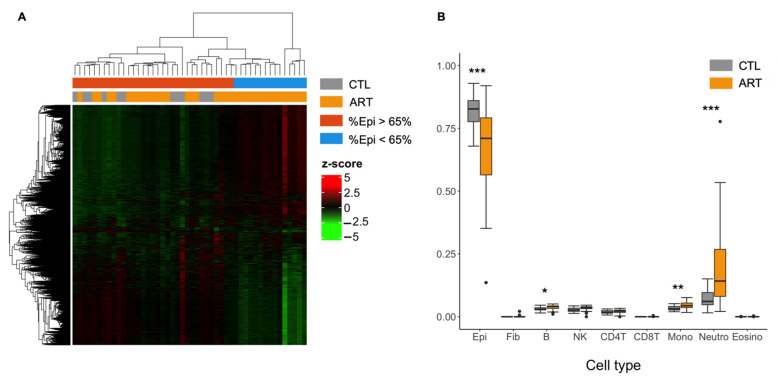
DMR analysis and the need to adjust for cell-type fractions. (**A**) Heatmap of differentially methylated regions (FDR < 0.05) between control and ART children in buccal cells without adjusting for cellular composition with hierarchical clustering of beta-levels (center scaled beta-values = z-scores). Epi = Epithelial buccal cells. Each row represents one of the 20,486 DMRs associated with ART. Each column corresponds to one sample. Dendrograms show how the samples and DMRs are independently clustered. The grey and orange header refers to the conception group to which children belong (grey = CTL, orange = ART). The blue and red header refers to the proportion of epithelial cells in each sample (blue = less than 65% epithelial cells, red = more than 65% epithelial cells). (**B**) Cellular proportions of buccal swab samples estimated by HEpiDISH between control and ART groups. Epi = Epithelial buccal cells, Fib = Fibroblasts, B = B cells, NK = Natural killer cells, CD4T = CD4T+ T cells, CD8T = CD8T+ T cells, Mono = Monocytes, Neutro = Neutrophils, Eosino = Eosinophils. * *p* < 0.05 ** *p* < 0.01 *** *p* < 0.001 (Student’s *t*-test).

Similar to the previous study, we conducted the first EWAS without adjusting for cell fractions in our model, assuming buccal smear samples were homogenous in our cohort. From all the probes tested, 17.1% showed differential methylation between ART and the control group (CTL) (FDR < 0.05) and 9.8% were differentially methylated with a Δβ > 5%. In total, 20,486 differentially methylated regions (DMRs) were found, 8527 of which contained at least one probe with Δβ > 5. Gene Ontology revealed that hypomethylated DMR genes were prevalent in immune biological pathways and hypermethylated DMR genes were prevalent in the epidermis processes. We performed a hierarchical clustering of DMRs (Figure 1A), which failed to properly separate CTL (in grey) from ART samples (in orange). It is likely that inappropriate data preprocessing was performed and that additional confounders, such as cell composition or the presence of outliers that we did not account for, had biased the differential methylation analysis (Figure 1A).

Using these observations, we estimated the cell proportions of buccal swab samples with a recent and accurate reference-based cell-type deconvolution method [46]. Surprisingly, from the DNA methylation profile estimations, predicted epithelial cell proportions ranged from 68.0% to 93.0% (mean = 81.6 ± 7.3%) for the control group whereas the ART group ranged from 13.6% to 92.0% (mean = 66.8 ± 17.0%) (Figure 1B). The ART group presented a significantly lower proportion of estimated epithelial cells (*p* = 0.00014) and a higher proportion of estimated neutrophils (*p* = 0.00016, mean control = 7.2 ± 4.1% vs. mean ART = 19.3 ± 16.1%). There was a strong negative correlation between epithelial cell and neutrophil proportions (r = −0.98) (Supplementary Materials File S3). To a lesser extent, there was also a difference in the estimated cellular fraction of monocytes (*p* = 0.0061) and B cells (*p* = 0.024) in our samples, and it was higher in the ART group. Similar to a previous study [63], estimates of CD8T cells, eosinophils, and fibroblasts were nearly null for all samples (Supplementary Materials File S4).

We finally noted that differentially methylated regions were biased by extreme ART samples that display abnormally low proportions of epithelial cells (Figure 1A). The observed differences are not related to the season of sample collection.

### 3.2. Impact of Mode of Conception, Type of Culture Medium, and Method of Fertilization at the CpG Level

**Figure 2:**
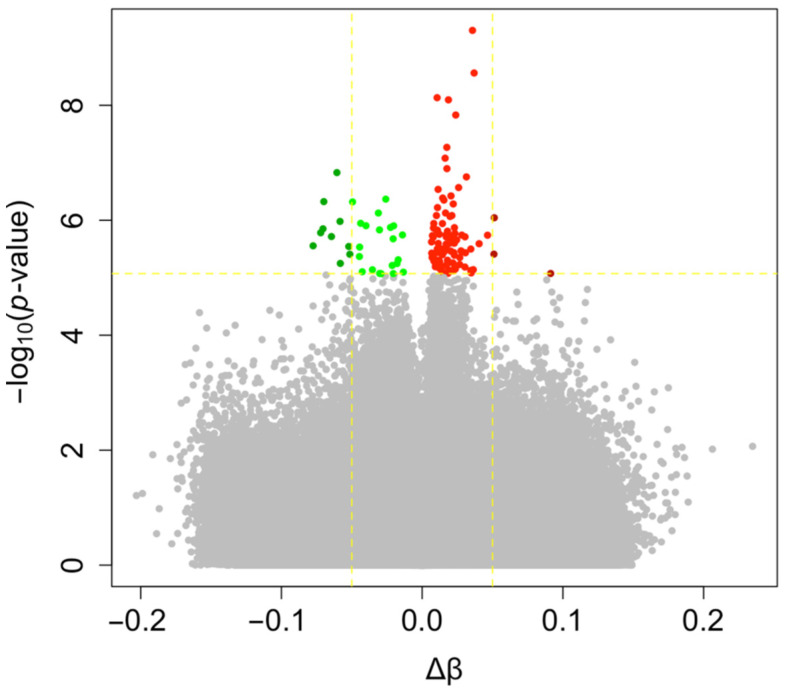
Volcano plot of DNA methylation changes with ART. Each point represents one of the 740,869 probes tested with mean differences in DNA methylation between ART and naturally conceived groups on the x-axis and −log10 of the unadjusted *p*-value from the moderated *t*-test computed with limma on the y-axis. Probes highlighted in green and red are respectively hypo- (Δβ < 0) and hyper-methylated (Δβ > 0) with ART (FDR < 0.05). Probes in dark green and dark red are those displaying Δβ > 5% (FDR < 0.05).

**Figure 3:**
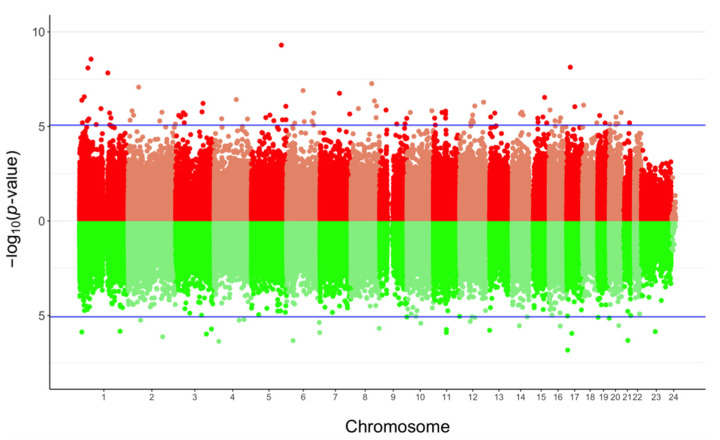
Bidirectional Manhattan plot of the genome-wide DNA methylation analysis of ART-conceived children compared to controls. Each point represents one of the 740,869 probes tested with their chromosomal location on the x-axis and −log10 of the unadjusted p-value from the moderated *t*-test computed with limma on the y-axis. Lines in blue separate probes that surpassed the FDR cut-off. The upper graph corresponds to hyper-methylated sites (in red) whereas the lower represents hypo-methylated sites (in green).

**Figure 4:**
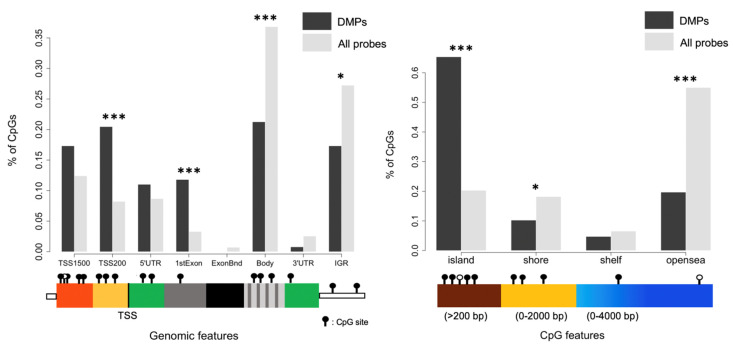
Distribution of DMPs across genomic and CpG regions. Significance was assessed with Chi-squared tests. * *p* < 0.05 *** *p* < 0.001. TSS = Transcription Start Site, IGR = Intergenic region.

In agreement with these previous observations, we performed a second analysis taking into account cellular fractions in our linear model. First, the average methylation level of the 740,869 probes tested was similar between ART-conceived children and controls (0.529 vs. 0.526, *p* = 0.221). Nonetheless, 127 differentially methylated positions (DMPs) were identified at FDR < 0.05 and 13 showing Δβ > 5% with ART (0.02% of all the probes tested); none of the probes exceeded a 10% methylation difference between the ART and naturally conceived groups (largest effect size: 0.091) (Figure 2; Supplementary Materials File S5). The majority of DMPs were hypermethylated (75.6%) in ART children compared with non-ART children (Figure 2) and they were scattered throughout the entire genome with no apparent preferential genomic position (Figure 3). DMPs were preferentially found in the 1st Exon and TSS200 and largely in CpG islands (Figure 4). The 20 top-ranked DMPs are shown in Table 1 and Supplementary Materials File S6.

We did not observe any DMPs in the ART group between children born via IVF or ICSI. Similarly, in vitro culture medium analysis did not reveal any DMPs between the Global and SSM groups.

### 3.3. Impact of the Mode of Conception, Type of Culture Medium, and Method of Fertilization at the Region Level

**Figure 5:**
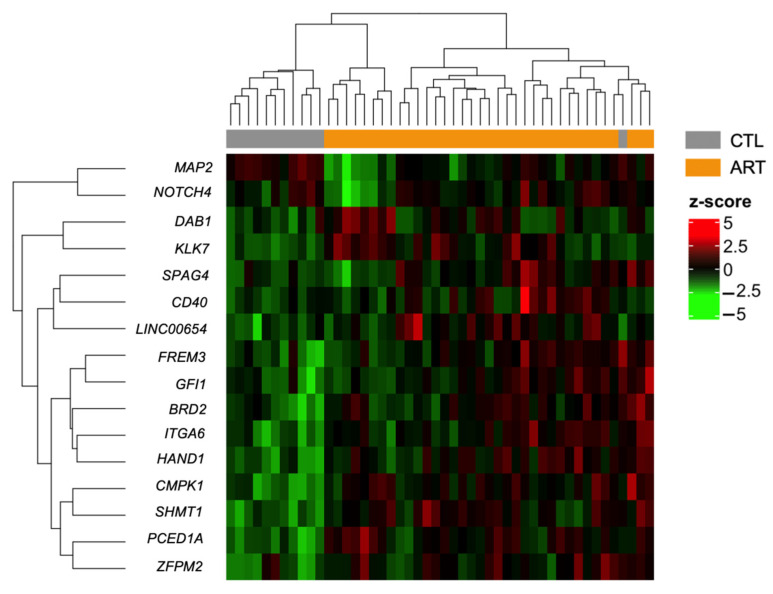
Heatmap of differentially methylated regions (FDR < 0.05) between control and ART children in buccal cells after adjusting for cellular composition with hierarchical clustering of beta-levels (center scaled beta-values = z-scores). Each row represents one of the 16 DMRs associated with ART. Each column corresponds to one sample. Dendrograms show how the samples and DMRs are independently clustered. The grey and orange header refers to the conception group to which children belong (grey = CTL, orange = ART).

We decided to focus our downstream analysis on DMRs because they may contain more biological information and limit redundancy among the DMPs as neighboring CpGs are often highly correlated [64]. In this study, DMRs are regions that contain at least two DMPs (FDR < 0.05). We identified 16 DMRs (Table 2; Supplementary Materials File S7 and S8), none of which were contained inside gene promoters. The majority of DMRs were hypermethylated, and only one was hypomethylated. All of them had a mean difference in methylation of <10% and only 2 had a mean difference >5%. Hierarchical clustering was managed properly to separate ART and non-ART groups (Figure 5).

We performed the Gene Ontology enrichment test on DMPs, but we did not find any significant ontology. Moreover, we tested our list of DMR genes in the DisGeNET database [52] in order to find if differentially methylated genes were known to be related to diseases. *ZFPM2* displayed a very high association score with tetralogy of Fallot (TOF), which is the most common form of congenital heart disease (Supplementary Materials File S9).

We did not observe any DMRs between the ART and CTL groups, ICSI and IVF groups, and Global and SSM groups.

A few studies have attempted to assess epigenome-wide effects in ART-born children. There were no DMPs or DMRs common between our study and two others performed in childhood and adolescence to date [40,61]. When we compared our results with the results of published work, regardless of the tested tissue and the age of the participants, we found six genes containing at least one DMR in common with a recent study on cord blood neonates [35] but not at the same genomic coordinates (*SHMT1*, *DAB1*, *ITGA6*, *ZFPM2*, *FREM3*, *KLK7*). We also found two genes containing one DMP in common with a study on neonates by Novakovic et al. [41]: *KAZN* and *ACTR3B*.

## 4. Discussion

To date, this is the only study that has performed an epigenome-wide analysis of ART outcomes in childhood assessing the impact of the culture medium in which ART children were conceived. The effects of different culture media have already been assessed in humans but only in imprinted regions and in placenta tissues [27]. This is also the second attempt to map epigenome-wide variation induced by ART in childhood [40].

For the first step, we carried out a genome-wide DNA methylation analysis to test for differences in the methylation profile of ART- and naturally conceived children. Indeed, concerns about the epigenetic safety of ART in the beginning of the century have not yet been totally set aside [65]. Even if long-term health follow-up of ART children is reassuring [6], epigenome-wide association studies of ART status have found inconsistent and conflicting results. This led us to pursue new insights about potential modifications in the methylome of ART children. We found here a small number of DMPs in the genome of ART children. In addition, our findings suggest the Δβ of DMPs and DMRs we found between ART and control groups at childhood mostly remain low (<5%). We are not able to fully appreciate to what extent a small variation in DNA methylation can affect gene expression, though it has been shown that small-sized effects observed in environmental studies can have big outcomes [66].

Intriguingly, we found DMR in *ZFPM2* which is a gene highly related to Tetralogy of Fallot (TOF) in the DisGeNET database and *ZFPM2* promoter hypermethylation has been found in TOF patients [67]. Moreover, *HAND1* and *NOTCH4*, in which we found DMR, display abnormal methylation patterns of their promoter regions in TOF patients [68,69]. Interestingly, TOF has been shown to be more prevalent in the ART-conceived population (adjusted OR 2.4, 95% CI 1.5–3.7) [70] even if an up-to-date meta-analysis did not find any increased risk of this congenital heart defect [71]. The etiology of TOF remains poorly understood, but recent studies suggest that an aberrant epigenetic status may play an important part in the development of this heart defect [72]. None of the DMRs we found in this study were located inside promoter regions though and should have a limited phenotypic impact. As a result, we can say that the methylome in ART children is not globally altered, so it may not affect global health until adulthood [73]. One-off changes in DNA methylation in imprinted regions cannot be excluded in ART children [28,74], but their functional relevance at a later age is still unknown.

Another key point of our research is that we were able to test whether the mode of in vitro fertilization (IVF or ICSI) or the use of differing embryo culture media are associated with differential DNA methylation outcomes. In our study, no DMPs or DMRs were found between the IVF and ICSI groups, which remains consistent with other epigenome-wide studies that did not find an effect relative to the conception method [60,75]. It is regrettable that this has been very little tested in EWAS. In fact, differences according to the use of IVF or ICSI were previously highlighted in LINE-1 in placental tissues [34,76] and imprinted genes *SNRPN* in children’s buccal cells [74], *PEG1*/*MEST* in cord blood [77], *H19* CTCF6 in children’s buccal cells [78], and *PLAGL1* in cord blood [79]. Penova-Veselinovic et al. [61] also noted four CpGs were differentially methylated between IVF and ICSI after multiple correction, but this association did not persist when correcting for additional confounders, such as the type of embryo transfer. Different assisted reproductive techniques thus may not induce considerable discrepancies between ART-conceived neonates, but we advise future studies to test for differences between IVF and ICSI in order to further explore this field.

Similarly, the use of distinct culture media (Global and SSM) was associated with discrepant developmental profiles in children [42]. However, both here and previously [28], we did not find any DMP or DMR between the Global and SSM culture medium groups in the same cohort. It is important and reassuring to notice that whereas SSM culture medium was significantly underperforming in early embryo development and pregnancy rates as compared with the Global medium, no epigenetic differences were found in large-scale analyses in children thus conceived. ART procedures’ specificities (i.e., IVF or ICSI, culture medium) tested in our cohort did not affect the methylome, and ART differences may thus come from the intrinsic biological features of gametes, ovarian hyperstimulation, or the use of the technique itself rather than its conditions [80,81]. Indeed, ART interferes at a time of intense physiological epigenetic reprogramming, i.e., during gametogenesis and early embryogenesis, when cells are highly sensitive to their environment. Efforts must be maintained to identify and assess the origins of epigenetic abnormalities detected in ART-conceived individuals in order to further improve ART procedures and reduce the potential health risks.

We compared our study with previous EWAS conducted in ART individuals, and again we found few gene correspondences. To date, no other EWAS was conducted on buccal cells and two were performed at childhood or adolescence. Penova-Veselinovic et al. [61] found no differences in adolescent blood methylome while Yeung et al. [40] found one DMR in children blood samples, but it was located in imprinted gene GNAS. We decided to perform a pan-tissue cross-over comparison of DNA methylation and ART studies whatever the age, which was motivated by the fact that ART could influence identical genes across different tissues and these modifications might be persistent during lifespan. The only similarities we found were with Novakovic et al. [41] (two genes containing at least one DMP in common) and Chen et al. [35] (six genes containing at least one DMR in common). We focused on buccal cells, which have less cell variability than blood and may be a more informative tissue for non-blood-based diseases in EWAS [82]. We attempted to link the DMPs we found to phenotypes thanks to the EWAS catalog [54] and the EWAS atlas [53]. Unfortunately, far too few EWAS were carried out on buccal cells, and they mostly focused on smoking, breastfeeding, and alcohol consumption, which restricts disease association.

The unique nature of our study arises from the fact that we assessed methylome in childhood (7.7 ± 0.7 years old), which has rarely been done on a genome-wide scale before, and almost all existing studies have focused on newborns. In addition, the age range of our cohort of children is very narrow, ensuring robust epigenetic assessments and comparisons. Indeed, Horvath established that DNA methylation was age dependent [83]. However, as DNA methylation is a dynamic process throughout life, it would be interesting to follow this cohort to reassess the methylome later, in the teenage years or adulthood, to fully understand how and if epigenetic variations induced by ART could be detrimental.

Finally, we found that the buccal swab samples of ART children presented a distinct composition profile. Based on estimations, immune cells were more prevalent, which is a possible biological characteristic of inflammation. More particularly, a very high proportion of neutrophils was found in ART samples compared to the control group. In line with this finding, perturbations in immune processes have already been described in the ART conceptus. Zhang et al. [84] first related differentially expressed genes implicated in the immune response in ART-treated placentas. Recently, Chen et al. [35] suggested that epigenetic modifications induced by ART could affect the immune system. This phenomenon has also been underlined in mouse experiments. Indeed, in vitro fertilization techniques dysregulated genes encoding proteins that play a role in immune regulation in placental tissues [85]. A study in ART-conceived mice also revealed disturbances in the TH1/TH2/TH17 balance [86], while another highlighted an altered helper T cell-mediated immune response [87]. It would be worth assessing whether the immune imbalance we observed in the cells of ART children shifts with age. If these observations are further confirmed by new studies, it would be interesting to explore the relation between the proportion of immune cells in the oral cavity, the epigenetic modifications potentially induced by ART, and a prospective predisposition to immune disorders.

To date, there are still gaps in our knowledge about the link between ART procedures and epigenetic variations observed in ART-conceived individuals on the epigenome-wide scale in humans. The one study that attempted to map global epigenetic changes following ART in early pregnancy by studying chorionic villus sampling did not find differences between ART and natural conception methods [58]. It has been suggested that abnormal epigenetic regulation during early embryogenesis could be a cause for abnormal placentation and may thus be the source of developmental abnormalities [88,89]. In addition, we still have a missing link between the epigenetic variation observed with ART and the disease phenotype. In our cohort, all children were born healthy and have not reported medical issues to date. Forming cohorts of individuals with identified medical conditions potentially linked to ART could be of great interest to investigate whether ART-induced epigenetic modifications could be responsible for these underlying disorders.

There are some limitations to our findings. Regarding the sample size, the statistical power of our epigenome-wide results can be debated. According to the a priori power calculations guidance in Mansell et al. [90], we would only detect 50% of all EPIC array sites with a mean difference of 5% with >50% power with 36 cases samples. Additionally, we decided to separate the ART group to analyze the fertilization method (IVF or ICSI vs. CTL) or the culture medium effect, which reduces the reliability of these sub-group analysis conclusions. It is, however, necessary to consider the remarkable homogeneity in our tested individuals (born in the same IVF center, ensuring the same lab conditions, at the same period, and very close in age). We can also ensure gestational age at birth between the two groups was not different between the ART and CTL groups. However, early life socioeconomic status could also be associated with specific DNA methylation patterns [91]. Unfortunately, this information was non-exhaustively recorded in our study to be assessed. To date, epigenome-wide association studies of ART effects suffer from great heterogeneity in sample size and ART procedures, which has resulted in discrepancies. Further studies of this kind would be welcome to address the growing problem of the origin of ART-induced epigenetic modifications.

## 5. Conclusions

Overall, our findings suggest that there are modest DNA methylation differences between naturally conceived and ART-conceived children and their functional relevance in adult tissue is unknown. Additionally, we highlighted that the use of different culture media and different techniques (ICSI or IVF) is not associated with DNA methylation variations across our cohort. Even if DNA methylation modifications in imprinted regions have been reported in relation to ART, the conclusions supported by our study tend to demonstrate additional modifications are not widespread across the entire genome. In accordance with epidemiological studies, our data are reassuring about the potential epigenetic side effects of ART in childhood. However, until now, too few studies have assessed the safety of ART on an epigenetic level. In future, it will be important to continue conducting epigenome-wide association studies in ART-conceived individuals, especially at various ages.

## Figures and Tables

**Table 1:** Top 20 differentially methylated positions associated with ART.

Probe ID	*p*-Value	Δβ	Genomic Feature	CpG Feature	Gene
cg25587535	0.0004	0.036	TSS200	island	*HAND1*
cg26311208	0.0010	0.037	TSS200	island	*DAB1*
cg18958584	0.0015	0.011	1stExon	island	*SHMT1*
cg24877558	0.0015	0.019	TSS1500	island	*FOXJ3*
cg18788524	0.0022	0.024	1stExon	island	*SEC22B*
cg17154315	0.0067	0.018	5′UTR	island	*ZFPM2*
cg02079951	0.0088	0.016	Body	island	*ASB3*
cg00270497	0.0117	0.018	TSS200	island	*RIPPLY2*
cg13593809	0.0122	−0.061	Body	shore	*LOC101559451*
cg19767562	0.0130	0.031	Body	island	*TFR2*
cg04856657	0.0179	0.026	TSS200	island	*PNRC2*
cg23727043	0.0179	0.011	TSS1500	island	*ADAMTS7*
cg05700616	0.0197	−0.026	IGR	opensea	*PPARGC1A*
cg11857246	0.0197	0.015	5′UTR	island	*MAD2L2*
cg14427382	0.0197	−0.070	Body	shore	*LOC100294145*
cg16866373	0.0197	0.015	5′UTR	island	*CCN3*
cg19306866	0.0197	−0.049	IGR	opensea	*KRTAP6-2*
ch.4.113910337F	0.0197	0.020	IGR	opensea	*ANK2*
cg00243897	0.0203	0.022	TSS200	island	*HPD*
cg12110529	0.0223	0.011	IGR	island	*ZBTB38*

**Table 2:** Differentially methylated regions associated with ART procedures identified by DMRcate. FDR corresponds to the minimum Benjamini–Hochberg FDR-corrected p-value in the region after Gaussian kernel smoothing.

Location (hg19)	Number of Probes	FDR	Maximum Difference	Mean Difference	Gene	Genomic Feature
chr19:51486901-51487968	14	4.49 × 10^−22^	0.084	0.038	*KLK7*	covers exons
chr20:34204902-34205488	7	2.81 × 10^−20^	0.062	0.030	*SPAG4*	covers exons
chr20:5485144-5486007	7	2.86 × 10^−20^	0.127	0.094	*LINC00654*	overlaps exon upstream
chr5:153857468-153858102	7	1.10 × 10^−16^	0.038	0.011	*HAND1*	overlaps exon upstream
chr20:44746392-44747351	9	2.19 × 10^−12^	0.103	0.064	*CD40*	covers exons
chr1:92949813-92950575	20	1.34 × 10^−11^	0.028	0.007	*GFI1*	inside intron
chr2:173292579-173292636	2	8.09 × 10^−11^	0.017	0.013	*ITGA6*	inside exon
chr4:144621270-144621385	3	1.34 × 10^−10^	0.017	0.013	*FREM3*	inside exon
chr1:59012392-59012820	11	1.84 × 10^−10^	0.035	0.001	*DAB1*	overlaps exon upstream
chr1:47799827-47800167	3	3.11 × 10^−10^	0.015	0.011	*CMPK1*	inside intron
chr20:2820742-2821472	14	8.23 × 10^−10^	0.019	0.008	*PCED1A*	covers exons
chr8:106331160-106331166	2	9.47 × 10^−10^	0.018	0.008	*ZFPM2*	inside exon
chr2:210444075-210444270	6	1.17 × 10^−09^	−0.067	−0.043	*MAP2*	inside intron
chr17:18266764-18266775	2	1.51 × 10^−09^	0.011	0.007	*SHMT1*	inside exon
chr6:32164503-32164801	7	1.88 × 10^−09^	0.053	0.034	*NOTCH4*	overlaps exon downstream
chr6:32939777-32940054	10	1.88 × 10^−09^	0.020	0.004	*BRD2*	inside exon

## Data Availability

The datasets generated during the current study are available in NCBI’s Gene Expression Omnibus under accession number GSE150901.

## Supplementary Materials

The following are available online at www.mdpi.com/article/10.3390/genes12121912/s1, Supplementary File S1: Characteristics of participating children, Supplementary File S2: Estimated Singular Value Decomposition of methylation sample analysis summary, Supplementary File S3: Correlation between estimated epithelial buccal cells fraction and other immune cells fraction (Cor: Pearson correlation coefficient), Supplementary File S4: Estimated cell proportions for each sample, Supplementary File S5: All DMPs with ART, Supplementary File S6: Dotplot of DNA methylation for control and ART samples for the top 20 top-ranked DMPs, Supplementary File S7: Dotplot of mean DNA methylation for control and ART samples for the 16 DMRs, Supplementary File S8: All DMRs with ART, Supplementary File S9: Gene-Disease heatmap from the DisGeNet database for gene containing DMRs.
