# Supplementary figures and images for: Genome-Wide Analysis of DNA Methylation in Buccal Cells of Children Conceived through IVF and ICSI
Source: Genes (Basel). 2021 Nov 28;12(12):1912. doi: 10.3390/genes12121912 (PMC8701402; doi:10.3390/genes12121912)

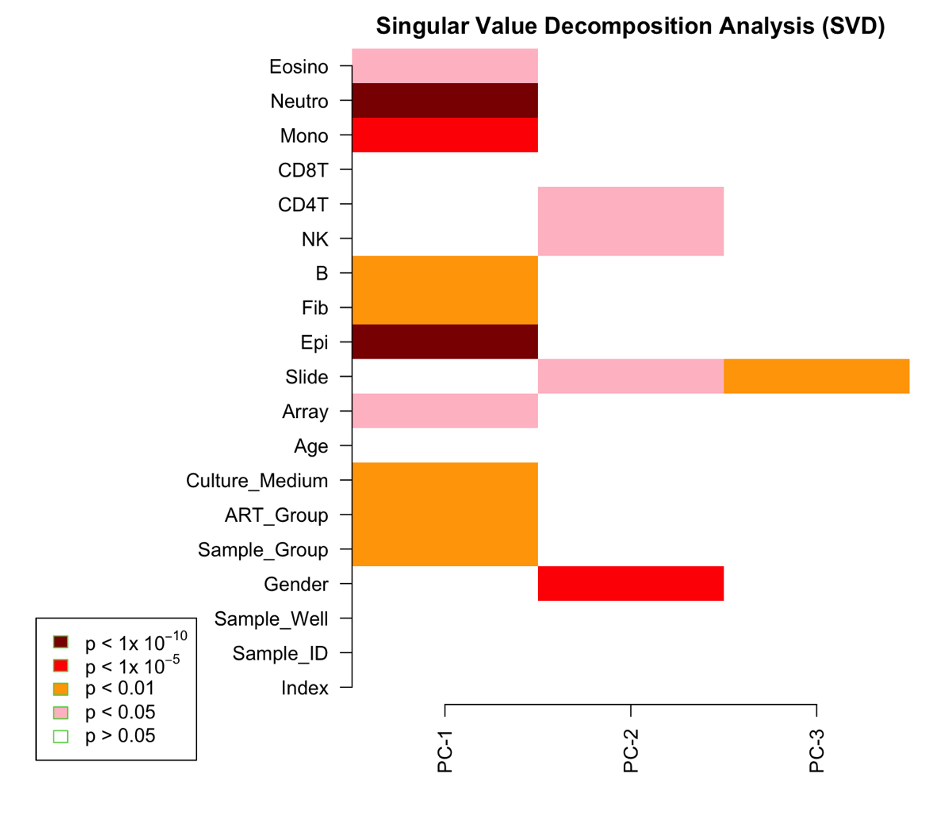

Supplement: Supplementary file 1 [file genes-12-01912-s001.zip › Supplementary File S2.tiff]

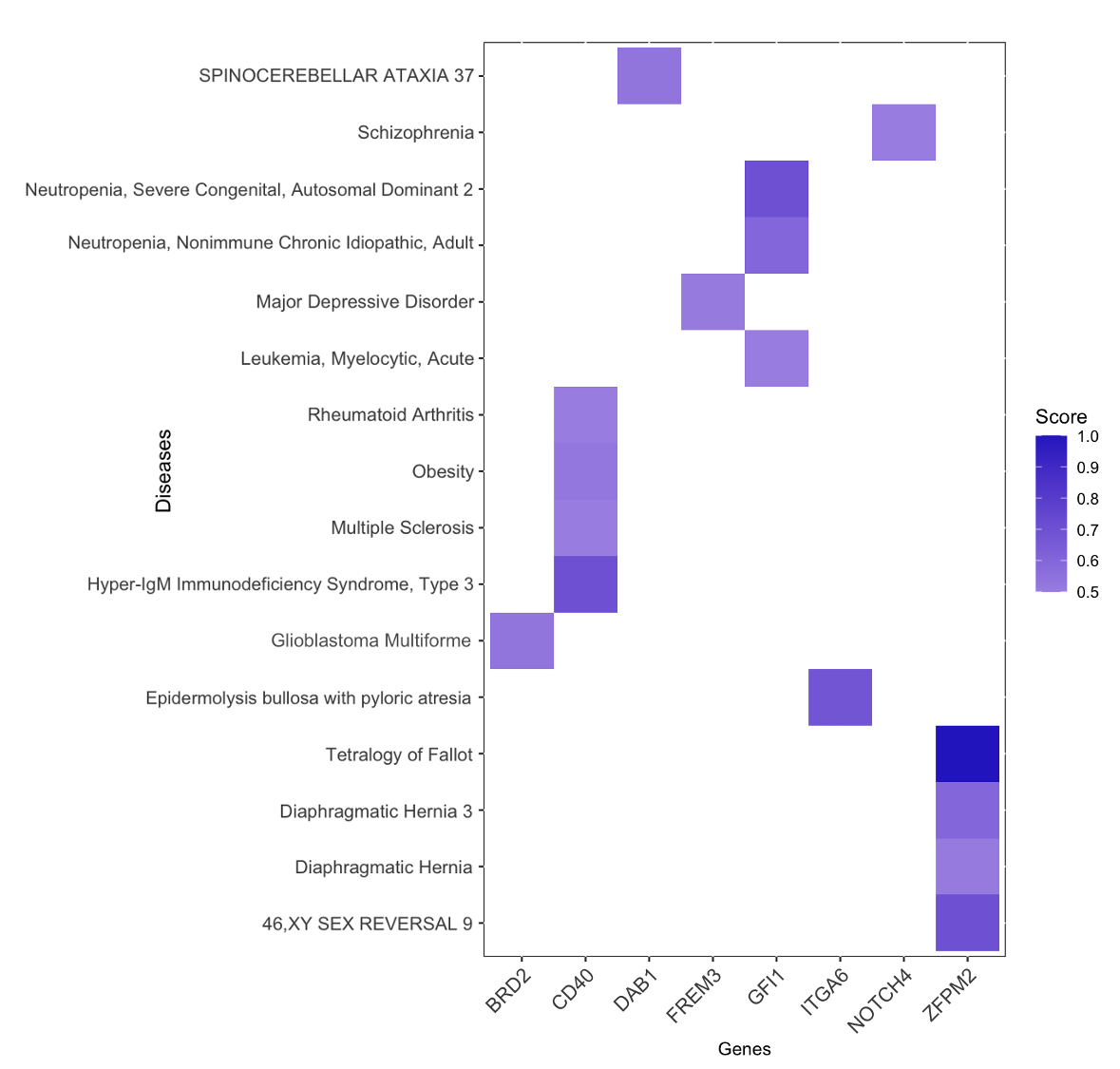

Supplement: Supplementary file 1 [file genes-12-01912-s001.zip › Supplementary File S9.tiff]
